# Prognostic Evaluation Tools to Facilitate Advance Care Planning in Two Older Patients With Terminal Cancer: A Report of Two Cases

**DOI:** 10.7759/cureus.78583

**Published:** 2025-02-05

**Authors:** Toshihiro Yamagata, Tomoya Oizumi, Shigeto Mashiko, Kaori Koyama, Katsutoshi Furukawa

**Affiliations:** 1 Division of Geriatric and Community Medicine, Faculty of Medicine, Tohoku Medical and Pharmaceutical University, Sendai, JPN; 2 Division of Supportive Medicine and Care for Cancer, Faculty of Medicine, Tohoku Medical and Pharmaceutical University, Sendai, JPN

**Keywords:** advance care planning, cancer, life support, prognostic evaluation tool, terminal care

## Abstract

Palliative prognostic index (PPI) is developed and utilized to assess the prognosis of terminally ill patients. Understanding the life expectancy is critically important for patients and their families. We present two terminally ill cases due to cancer in which we used prognostic evaluation tools during advance care planning (ACP) among patients, families, and medical staff. In the first case, the patient and her family initiated an ACP early in her diagnosis. The unfavorable prognoses indicated by PaP and PPI supported and reaffirmed their decision for no intensive treatment or life support, allowing her to pass away peacefully at home. The second case involves a family initially reluctant to accept the patient's ACP, which refused intensive therapy or life support for malignant tumors. However, the family understood and accepted the patient’s initial ACP through careful discussions utilizing these prognostic tools. These cases highlight the importance of using objective prognostic prediction tools to support clinical judgments, particularly in the terminal stages of malignant tumors.

## Introduction

This report discusses the successful application of the palliative prognostic (PaP) score [1] and the palliative prognostic index (PPI) [2] in evaluating and supporting advance care planning (ACP) for two cancer patients with cancer, highlighting the challenges and necessity of objective prognosis assessment. ACP has become critically important as many people live longer lives, and making critical decisions can be challenging if they are affected by dementia or terminal illnesses [3]. ACP is dynamic and can alter throughout a person's life and change in decisions as diseases progress. Therefore, ACP should be viewed as a process involving ongoing dialogue among patients, their families, and medical professionals. Decisions such as how long to continue life support in the terminal stage of cancer are often complex and distressing for both patients and families. Patients diagnosed with terminal-stage cancer frequently exhibit non-specific symptoms across various organs, necessitate hospitalization in non-specialized departments, and face challenges due to limited treatment options. In such scenarios, accurately assessing the prognosis and implementing ACP are vital for individuals and their families [4].

## Case presentation

Case 1

A female patient in her 80s experienced whole-body pain for one month before being admitted, which severely restricted her mobility and necessitated emergency transportation. Plain whole-body CT scans revealed diffuse sclerotic changes throughout her bones, suggesting bone metastasis from an unknown primary malignancy and prompting further tests. A bone marrow biopsy confirmed adenocarcinoma, although the primary site remained unidentified. The patient had high serum potassium levels, serum creatinine, and blood urea nitrogen, indicating renal failure. The patient and her family had previously established an ACP, deciding against aggressive treatment and a full work-up in the event of a malignant disease. In addition to the Karnofsky performance status (KPS) of 10, her PaP score was 12.7, predicting a less than 30% chance of survival beyond 30 days [1]; her PPI was 4.5, suggesting a life expectancy of 13-24 days [2]. Given the limited time, discussions were repeatedly held with the patient and her family from the time of admission to refine her ACP. Following her wish to spend the remaining time at home, her primary physician facilitated her discharge with psychological support from medical staff and her family. The physician carefully explained the prognosis to the patient and her family, who understood and peacefully agreed to continue the ACP to allow her to pass away at home. Shortly after returning home, she experienced a sudden decline in consciousness and difficulty breathing, leading to her death 47 minutes after arrival.

Case 2

A female patient in her 70s had been experiencing general malaise and difficulty with oral intake for one month before admission, during which time a neck mass appeared and enlarged. A lymph node biopsy confirmed malignant lymphoma, leading to her readmission to the hematology department for treatment initiation. Unfortunately, her condition deteriorated rapidly. Initially, the patient's ACP made by herself stated, "Do not attempt resuscitation in the case of malignancy or incurable disease," but her family had not been involved in this decision. Her PaP score was 11.4, including a KPS of 10, predicting a less than 30% chance of survival beyond 30 days [1]; her PPI was 4.5, suggesting a life expectancy of 13-24 days [2]. On the eighth day after admission, the primary physician thoroughly explained her condition to her common-law husband. Despite his initial struggle to accept the rapid progression of her illness and his inability to make decisions, the clear explanations using the PaP and PPI scores enabled him to discuss and establish a revised ACP, agreeing to forego further intensive life-support therapies or care. She received minimal life-support measures, such as oxygen through a nasal cannula, and passed away quietly and peacefully; this allowed the family to understand and accept the situation.

The characteristics of the two cases are summarized in Table 1.

**Table 1:** Characteristics of the two cases PaP: palliative prognostic, PPI: palliative prognostic index

	Case 1	Case 2
Age	80s	70s
Gender	Female	Female
Disorder	Cancer of unknown primary origin	Malignant lymphoma
PaP score	12.7	11.4
Prediction by PaP score	<30% chance of survival beyond 30 days	<30% chance of survival beyond 30 days
PPI score	4.5	4.5
Prediction by PPI score	Life expectancy of 13-24 days	Life expectancy of 13-24 days

## Discussion

In palliative care settings, tools like the PPI and PaP can effectively predict patient outcomes [1,2,5]. The PaP score factors include clinical prediction of survival, KPS, symptoms like anorexia and dyspnea, total white blood count, and lymphocyte count [1]. The provided PPI assesses KPS, oral intake, edema, dyspnea at rest, and delirium [2]. While PaP scores are more accurate for monthly prognosis evaluations, PPI scores are superior for weekly assessments. In both cases, these prognostic prediction tools played crucial roles in facilitating the ACP process and supporting the emotional well-being of the families. Prediction of life expectancy should be one of the most critical issues for patients with terminal illness and their families in palliative care. There are also other prognostic predicting tools, which are for geriatric [6], postsurgical [7], and advanced [8] malignant disorder patients. Accurate prediction of survival prognosis must be important for managing effective palliative care. To decide what medical intervention is appropriate for a patient, physicians should evaluate the benefits and disadvantages of each intervention based on survival prediction [9]. Appropriate prognostic diagnoses using prognostic evaluation tools can help patients and their families fulfill their final wishes. In addition to that, medical professionals can choose the best end-of-life care in accordance with evaluation tools and the patient’s wishes [10]. Newer predictive tools have been developed, such as the Japanese palliative oncology study prognostic index [11] and the objective predictive score [12]. Moreover, prognostic prediction tools and ACP should be employed not only in cases of terminal malignancies but also in those with dementia and geriatric syndromes [13]. Future medical practices should integrate objective prognosis prediction tools to support clinical judgment.

## Conclusions

We encountered two cases of terminally ill patients with malignant tumors where prognostic evaluation tools significantly aided the ACP process. These cases underscore the value of these tools in providing prognoses that facilitate ACP, particularly for older adults. Physicians should use these prediction tools not only for patients with malignant tumors but also for those experiencing geriatric syndromes to support their ACP.
